# Mr. Sergeant, on Vaccine

**Published:** 1800-12

**Authors:** Tho. Sergeant

**Affiliations:** Green Street, Grosvenor Square


### *To the Editors of the Medical and Phyfical Journal.*

Gentlemen,

Cases are, I believe, no longer requifite to prove the efficacy of the Cow-pox in preventing the Small-pox; and hence it is probable, the circumftances hereafter to be ftated may not be worthy of your infertion: I fhall however communicate the facts, leaving you to decide on the propriety of making them public.

On the 6th of September, I inferted the vaccine lymph, which had been fuffered to dry on the lancets, into the arms of an healthy child, about eight months old, who had been inoculated for the fame difeafe a few months previous, by another practitioner, without fuccefs; this infertion alfo failed, and I repeated the inoculation on the eighth day, immediately from the arm of another infant. The ufual appearances taking place in each arm, the patient went regularly through the difeafe. Now, there was a fifter fome months older, the inoculation of whom, the parents were defirous to defer till lymph might be obtained from the brother; but on the fixth day from the fuccefsful inoculation of the latter, the former had a plentiful eruption, which proved to be variolous. The difeafe of each proceeded proceeded regularly, and I frequently ſaw the children laying alternately in one cradle.

On the tenth day of the vaccine diſeaſe, I had the child who had been thus expoſed to the variolous contagion, brought to my houſe, and from him inoculated three other children, who alſo have gone regularly through the diſeaſe.

The former patient continuing in good health, and not having to this time received the variolous contagion, adds one very ſtrong caſe to the many already made known, to prove the preventive effects of Vaccine Inoculation. I am,

Gentlemen,

Your humble ſervant,

THO. SERGEANT.

*Green Street, Grosvenor Square,*

*October* 8, 1800.

